# Principles and mechanisms of regeneration in the mouse model for wound‐induced hair follicle neogenesis

**DOI:** 10.1002/reg2.38

**Published:** 2015-06-09

**Authors:** Xiaojie Wang, Tsai‐Ching Hsi, Christian Fernando Guerrero‐Juarez, Kim Pham, Kevin Cho, Catherine D. McCusker, Edwin S. Monuki, Ken W.Y. Cho, Denise L. Gay, Maksim V. Plikus

**Affiliations:** ^1^ Department of Developmental and Cell BiologyUniversity of California, IrvineIrvineCalifornia92697USA; ^2^ Sue and Bill Gross Stem Cell Research CenterUniversity of California, IrvineIrvineCalifornia92697USA; ^3^ Center for Complex Biological SystemsUniversity of California, IrvineIrvineCalifornia92697USA; ^4^ Department of Pathology and Laboratory MedicineUniversity of California, IrvineIrvineCalifornia92697USA; ^5^ UMR 967, Cellules Souches et Radiations, CEA – INSERM – Universités Paris 7 et Paris 11CEA/DSV/IRCM/SCSR/LRTS92265 Fontenay‐aux‐Roses CedexFrance

**Keywords:** Hair follicle, mouse, neogenesis, regeneration, skin, WNT, wound

## Abstract

Wound‐induced hair follicle neogenesis (WIHN) describes a regenerative phenomenon in adult mammalian skin wherein fully functional hair follicles regenerate de novo in the center of large excisional wounds. Originally described in rats, rabbits, sheep, and humans in 1940−1960, the WIHN phenomenon was reinvestigated in mice only recently. The process of de novo hair regeneration largely duplicates the morphological and signaling features of normal embryonic hair development. Similar to hair development, WIHN critically depends on the activation of canonical WNT signaling. However, unlike hair development, WNT activation in WIHN is dependent on fibroblast growth factor 9 signaling generated by the immune system's γδ T cells. The cellular bases of WIHN remain to be fully characterized; however, the available evidence leaves open the possibility for a blastema‐like mechanism wherein epidermal and/or dermal wound cells undergo epigenetic reprogramming toward a more plastic, embryonic‐like state. De novo hair follicles do not regenerate from preexisting hair‐fated bulge stem cells. This suggests that hair neogenesis is not driven by preexisting lineage‐restricted progenitors, as is the case for amputation‐induced mouse digit tip regeneration, but rather may require a blastema‐like mechanism. The WIHN model is characterized by several intriguing features, which await further explanation. These include (1) the minimum wound size requirement for activating neogenesis, (2) the restriction of hair neogenesis to the wound's center, and (3) imperfect patterning outcomes, both in terms of neogenic hair positioning within the wound and in terms of their orientation. Future enquiries into the WIHN process, made possible by a wide array of available skin‐specific genetic tools, will undoubtedly expand our understanding of the regeneration mechanisms in adult mammals.

## The hair follicle as the model for mammalian regeneration

The hair follicle (HF), a defining anatomical feature of all mammals, is an intricate mini‐organ composed of epithelial and mesenchymal cells that work in concert to generate a hair shaft. The HF's epithelial cells proliferate and differentiate to become a shaft, while its mesenchymal components, primarily the dermal papilla, function as the HF's signaling center. Anatomically, the HF can be divided into a permanent upper portion and a transient lower portion, and within the permanent portion of the HF lies its slow cycling stem cells, also known as bulge stem cells (Cotsarelis et al. 1990; Morris et al. 2004; Tumbar et al. 2004; Snippert et al. 2010). The progeny of these stem cells divide rapidly and generate all the lower HF's structures, including the hair shaft (Taylor et al. 2000; Morris et al. 2004). Hair shafts grow for a finite period of time, reflecting the underlying cyclical nature of physiological HF regeneration. The so‐called hair growth cycle consists of phases of active growth (anagen), involution (catagen), and relative quiescence (telogen) (Stenn & Paus 2001; Schneider et al. 2009).

**Figure 1:**
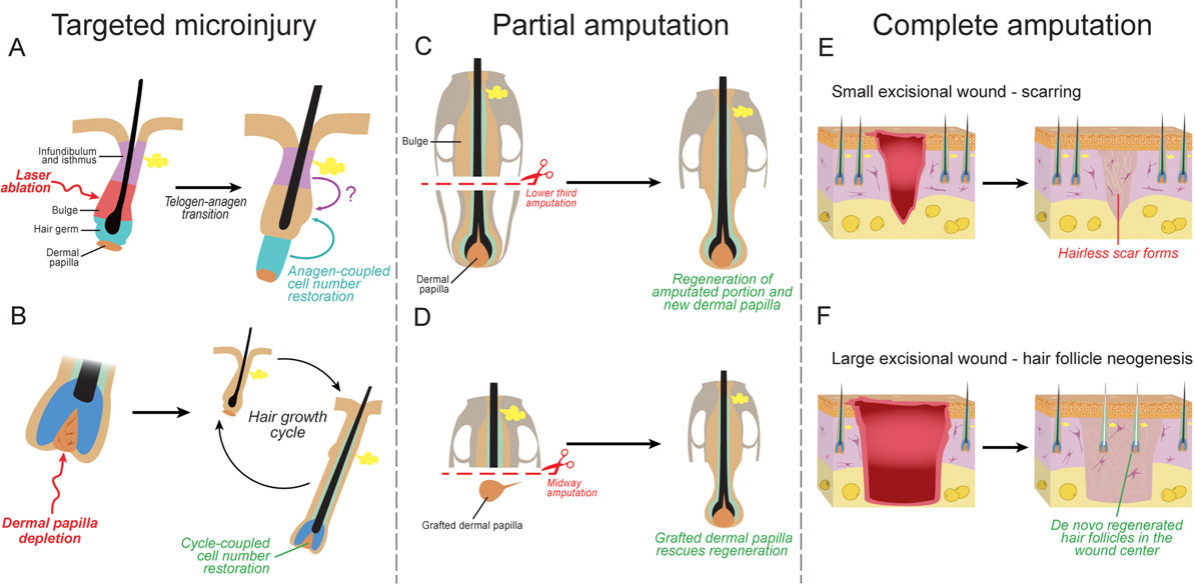
Injury types and regenerative responses by adult HFs. HFs can efficiently regenerate following micro‐injury, as well as partial and complete amputation. (A), (B) Micro‐injury of bulge stem cells in telogen HFs, such as by laser ablation, can be efficiently repaired from the neighboring epithelial progenitor populations in the hair germ and possibly isthmus. Genetic ablation of dermal papilla cells in anagen HFs can be restored from the surviving dermal papilla cells and/or via recruitment of the neighboring dermal sheath cells. (C), (D) Anagen vibrissa follicles efficiently regenerate following amputation of the lower third, which includes the entire dermal papilla. Midway (lower half) amputations can also regenerate; however, this requires transplantation of a new dermal papilla. (E), (F) HFs can regenerate de novo following large excisional skin wounding in adult mice. This regenerative phenomenon is known as wound‐induced hair follicle neogenesis (WIHN). WIHN does not occur in small excisional wounds.

While the HF displays prominent physiological regeneration, it also regenerates following injury. Indeed, the adult HF can efficiently “rebuild” after micro‐injury, partial amputation, and even complete amputation (Fig. 1), making it a valuable model for studying cellular and signaling mechanisms of injury‐induced regeneration in mammals.

## Injury types and regenerative responses

Broadly speaking, three types of injury can be recognized depending on the severity: (1) micro‐injury, when individual cells or small groups of cells are lost; (2) partial amputation, when part of the complex structure, with one or several distinct cell types, is destroyed; and (3) complete amputation, when the whole tissue with all its cells, including parenchyma and stroma, is lost. The regenerative mechanism in the first case may only require reshuffling of cell positions; however, complete amputation requires tissue regeneration from “scratch,” a process that may involve the formation of a blastema. Below, we briefly discuss HF regeneration responses after all three injury types (Fig. 1).

### Regeneration following micro‐injury

Regeneration following micro‐injury occurs within a largely preserved tissue architecture and a largely undisturbed complex signaling environment. Recently, the study of HF responses to such micro‐injuries became possible with the use of targeted laser ablation (Rompolas et al. 2013) and genetic cell ablation techniques (Hsu et al. 2011; Chi et al. 2013). With both techniques, a specific cell population can be selectively targeted and destroyed, and the cellular dynamics that follow can be studied using fate‐mapping approaches.

Micro‐injuries of HF epithelial stem cells are typically repaired efficiently via recruitment of nearby progenitors. When the HF bulge is laser‐ablated during telogen, the vacant niche is repopulated by the neighboring hair‐fated progenitors from the hair germ, and possibly from supra‐basal stem cells as well. As a result, damaged HFs can reenter the hair growth cycle (Rompolas et al. 2013) (Fig. 1A). Similarly, HFs repair and regenerate following partial depletion of bulge stem cells via inducible expression of diphtheria toxin fragment A (DTA) using a bulge‐specific *Cre* driver (Hsu et al. 2011). In this case, repair occurs from the remaining bulge stem cells that survive DTA depletion.

Micro‐injuries in the HF mesenchymal compartment can also be repaired. Partially DTA‐depleted dermal papilla can be restored over time as the HF cycles, such as through cell proliferation during early anagen (Chi et al. 2010) (Fig. 1B). Cells from the neighboring dermal sheath can also contribute to dermal papilla repair (Jahoda 2003; McElwee et al. 2003; Tobin et al. 2003; Rahmani et al. 2014). As a result, HFs can continue to cycle, although they may produce more diminutive hair shaft types (Chi et al. 2013). Interestingly, if dermal papilla depletion is more dramatic, leaving fewer than 10 cells, HFs cease to cycle and become arrested in telogen (Chi et al. 2013). Similarly, the dermal papilla cannot regenerate following its complete laser ablation during telogen, and subsequently HFs fail to regenerate (Rompolas et al. 2012). Taken together, repair of HF micro‐injuries mainly occurs using neighboring cells, which share a close lineage relationship and micro‐anatomical location with the lost cells.

Although not specifically investigated in the HF, repair following micro‐injury in mammals can involve cell type reprogramming, the mechanism referred to as “facultative stem cell” activation (Desai & Krasnow 2013). As the term implies, facultative stem cells under normal conditions are differentiated cells that take on a multipotent state following injury. Regeneration via facultative stem cells occurs only in special circumstances, such as when they share a close developmental origin with the missing cell type. For example, following toxin‐induced depletion of the liver's biliary epithelial cells, regeneration can occur via reprogramming of hepatocytes (Yanger et al. 2013; Yanger & Stanger 2014). In the lung, alveolar type 1 cells can be replaced through reprogramming of neighboring type 2 cells following selective type 1 cell ablation via hyperoxic injury (Desai et al. 2014). In the trachea, differentiated secretory cells can dedifferentiate and convert into new basal epithelial stem cells following genetic ablation of the endogenous basal stem cell population (Tata et al. 2013). Similarly, in the stomach corpus, differentiated secretory Troy+ chief cells can acquire stem cell properties and regenerate the entire crypt following genetic depletion of the primary crypt's stem cell compartment (Stange et al. 2013). Importantly, all these small‐scale regeneration responses do not rely on the formation of a blastema. Instead, they occur via repopulation of vacant anatomical niches, which supply the necessary signaling cues for proper repair.

### Regeneration following partial amputation

The anagen HF can also regenerate following amputation of its lower portion. HF responses to partial amputation have been a subject of extensive research in classic studies on the model of rat vibrissae (reviwed in Plikus 2014). When the lower third of the vibrissa HF, along with its dermal papilla, is amputated, repair occurs from the remaining upper portion (Fig. 1C). The amputated vibrissa HF regenerates a new dermal papilla and reenters anagen, suggesting restored functionality (Oliver 1966). This more profound regeneration is not limited to vibrissae but also occurs in human HFs, albeit with low efficiency (Jahoda et al. 1996). Regeneration of the dermal papilla appears to be a key factor that limits vibrissa regeneration after more extensive amputations. Although midway‐amputated vibrissa follicles generally fail to regenerate spontaneously, they can regenerate following dermal papilla grafting (Oliver 1967) (Fig. 1D). These observations suggest that lower, but not upper, dermal sheath cells can regenerate new dermal papilla because HFs fail to regenerate when the lower dermal sheath is completely removed. Furthermore, the ability of midway‐amputated HFs to regenerate also suggests that upper epithelial cells, which include bulge stem cells, are competent to regenerate the missing HF parts, provided that they receive instructive signals from the dermal papilla. Importantly, the occurrence of this phenomenon in rodent pelage HFs has not yet been definitively reported.

### Regeneration following complete amputation

Conventionally, partial amputation was thought to represent the upper limit of adult HF regeneration, and injuries that involved a more profound loss of HF structures, such as complete HF loss in excisional skin wounds, were considered irreparable. Indeed, skin wounds in adult mammals typically heal with scarring (Fig. 1E). However, in instances when excisional wounds are large (1 cm in diameter in mice), de novo HFs can regenerate in the wound's center—a phenomenon known as wound‐induced hair follicle neogenesis (WIHN) (Dann et al. 1941; Taylor 1949; Breedis 1954; Billingham & Russell 1956; Kligman & Strauss 1956; Billingham 1958; Kligman 1959; Brook et al. 1960; Mikhail 1963; Stenbäck et al. 1967; Ito et al. 2007; Seifert et al. 2012; Gay et al. 2013) (Fig. 1F). Considering that HFs form only once during embryonic development and normally do not do so in adult skin, WIHN is an example of embryonic‐like regeneration—a type of regenerative response rarely seen in mammals.

Embryonic‐like regeneration is prevalent in non‐mammalian vertebrates, and two principal regenerative responses can be distinguished based on the cellular mechanism: (1) regeneration from lineage‐restricted, tissue‐specific stem cells, and (2) regeneration via lineage reprogramming, such as in the process of dedifferentiation−redifferentiation. The former mechanism is observed upon mouse digit tip regeneration, another well‐known example of embryonic‐like regeneration in mammals (Lehoczky et al. 2011; Rinkevich et al. 2011; Takeo et al. 2013; Leung et al. 2014). Similarly, during limb regeneration in the axolotl *(Ambystoma mexicanum)*, skeletal muscle regenerates from preexisting Pax7+ muscle‐fated stem cells (Kragl et al. 2009; Sandoval‐Guzman et al. 2014). An example of embryonic‐like regeneration via cellular reprogramming is the regeneration of an amputated eye lens in newts and frogs, which occurs via reprogramming of the iris pigmented epithelial cells (Freeman 1963; Eguchi et al. 1974; Henry & Elkins 2001; Tsonis & Del Rio‐Tsonis 2004). Additionally, radial neuroglia cells can reprogram into muscle and cartilage during tail regeneration (Echeverri & Tanaka 2002), and dermal fibroblasts can reprogram into chondrocytes during limb regeneration in the axolotl (Kragl et al. 2009; Hirata et al. 2010).

The latter mechanism of regeneration is commonly associated with the formation of a blastema. Although the term “blastema” is deeply rooted in the context of Urodele regeneration biology, broadly speaking it defines a mass of proliferating multipotent progenitors at the site of amputation that serves as the cellular source for de novo regeneration (Hay & Fischman 1961; O'Steen & Walker 1961; Gardiner et al. 1986; Muneoka et al. 1986; Roensch et al. 2013). In Urodeles, the formation of the blastema requires the induction of a specialized epidermis known as the apical epithelial cap (Singer & Inoue 1964), which secretes a number of signaling morphogens including fibroblast growth factors (FGFs) (Christensen et al. 2002; Satoh et al. 2011), bone morphogenic proteins (BMPs) (Makanae et al. 2014), and Wingless‐Int (WNTs) (Ghosh et al. 2008; Shimokawa et al. 2013). Cellular reprogramming is thought to be one of the mechanisms by which blastema cells acquire multipotency (Satoh et al. 2008a; Satoh et al. 2008b; McCusker & Gardiner 2013). To date, reprogramming is yet to be confirmed for embryonic‐like regeneration in mammals; however, blastema‐like histological features have been noted during ear regeneration in the African spiny mouse (*Acomys*) (Seifert et al. 2012; Tanaka 2012). Below, we argue that the phenomenon of HF neogenesis in large skin wounds, in combination with the plethora of genetic tools available in *Mus musculus*, presents itself as a highly promising and tractable experimental model to further probe for blastema‐like regeneration in mammals.

## Basic features of the WIHN model

Although neither was recognized at the time as WIHN, de novo regeneration of HFs was first observed in adult rats by Dann et al. (1941) following excisional wounding and by Taylor (1949) following full‐thickness skin cryo‐injury. Several years later, the WIHN phenomenon was reported and explicitly recognized in rabbits by Breedis (1954) and Billingham & Russell (1956). Breedis (1954) wrote that following excisional wounding “functioning hair follicles and sebaceous glands appeared in the scars, sometimes in great profusion.” Similarly, Billingham & Russell (1956) reported that “with the production of these [neogenic] hairs the originally smooth scars may be said to have become transformed into a sort of ad hoc skin.” Additional studies confirmed WIHN in sheep (Brook et al. 1960), rarely in humans (Kligman & Strauss 1956; Kligman 1959), and once again in rats (Mikhail 1963) and rabbits (Stenbäck et al. 1967). Importantly, around the time of its discovery, the WIHN phenomenon was not universally accepted. After repeating full‐thickness wounding experiments in rabbits, Straile (1959) concluded that “uninjured follicles moved from the periphery into the wounds and repopulated them without evidence of a neoformation.” However, Billingham (1958) argued that “there can be little doubt that an interaction of epidermis and dermis is involved in initiating the development of hairs, and the process of hair neogenesis, as seen in wounds in adult rabbits, probably does not differ significantly from that which occurs normally in neonatal life.”

Surprisingly, these early accounts of the WIHN phenomenon went largely forgotten, and during the next four decades the prevailing dogma was that HFs form only once in ontogenesis—during embryonic development—and that skin wounds in adults inevitably heal into hairless scars. Only recently, following the landmark study by Ito et al. (2007), did the re‐discovery of the WIHN phenomenon in adult mice occur. Through careful observations and with the help of an array of genetic mouse tools, Ito et al. (2007) unequivocally confirmed that HFs in the wound's center regenerate de novo via a process that recapitulates normal embryonic hair morphogenesis. A series of recent studies describing the cellular and signaling aspects of hair neogenesis have dramatically raised awareness of the WIHN phenomenon, carrying it into the broader scope of stem cell biology and regenerative medicine (Fan et al. 2011; Sun et al. 2011; Seifert et al. 2012; Driskell et al. 2013; Fuchs et al. 2013; Gay et al. 2013; Myung et al. 2013; Nelson et al. 2013; Takeo et al. 2015). A study by Seifert et al. (2012) on hair neogenesis following autotomy‐like skin shedding in *Acomys* is of particular interest (also reviewed in Tanaka 2012; Seifert & Maden 2014). It demonstrates that WIHN can be an indispensable part of a natural adaptation against predation—spiny mice have very fragile skin that breaks easily, leaving large full‐thickness wounds that efficiently regenerate numerous HFs.

**Figure 2:**
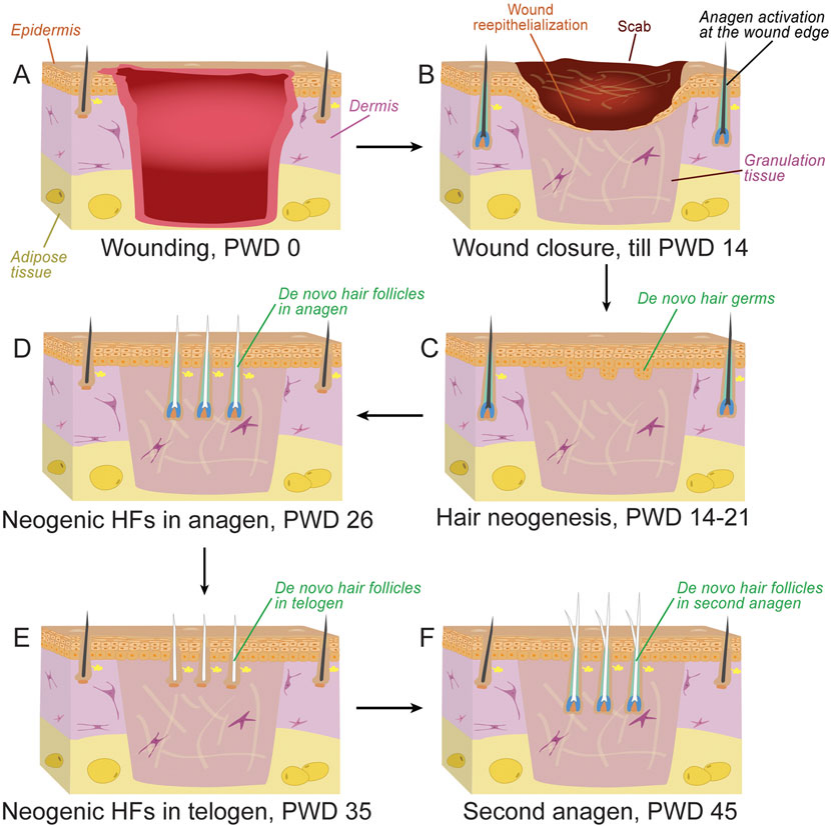
Timeline of hair follicle regeneration in the WIHN model. (A) Hair neogenesis in mice occurs in large excisional wounds equal to, or larger than, 1 × 1 cm. (B) The wound epithelializes and granulation tissue forms during early PWD0−14. (C) De novo hair placodes start to form around PWD14 and continue until approximately PWD19. (D) Newly formed HFs achieve full differentiation over the next 14−15 days (until approximately PWD33−34). (E), (F) Following a transient telogen phase (PWD35), de novo follicles reenter second anagen at around PWD45. Similar to normal HFs in the unwounded skin, de novo follicles in the wound center contain bulge stem cells and can cycle repetitively.

**Figure 3:**
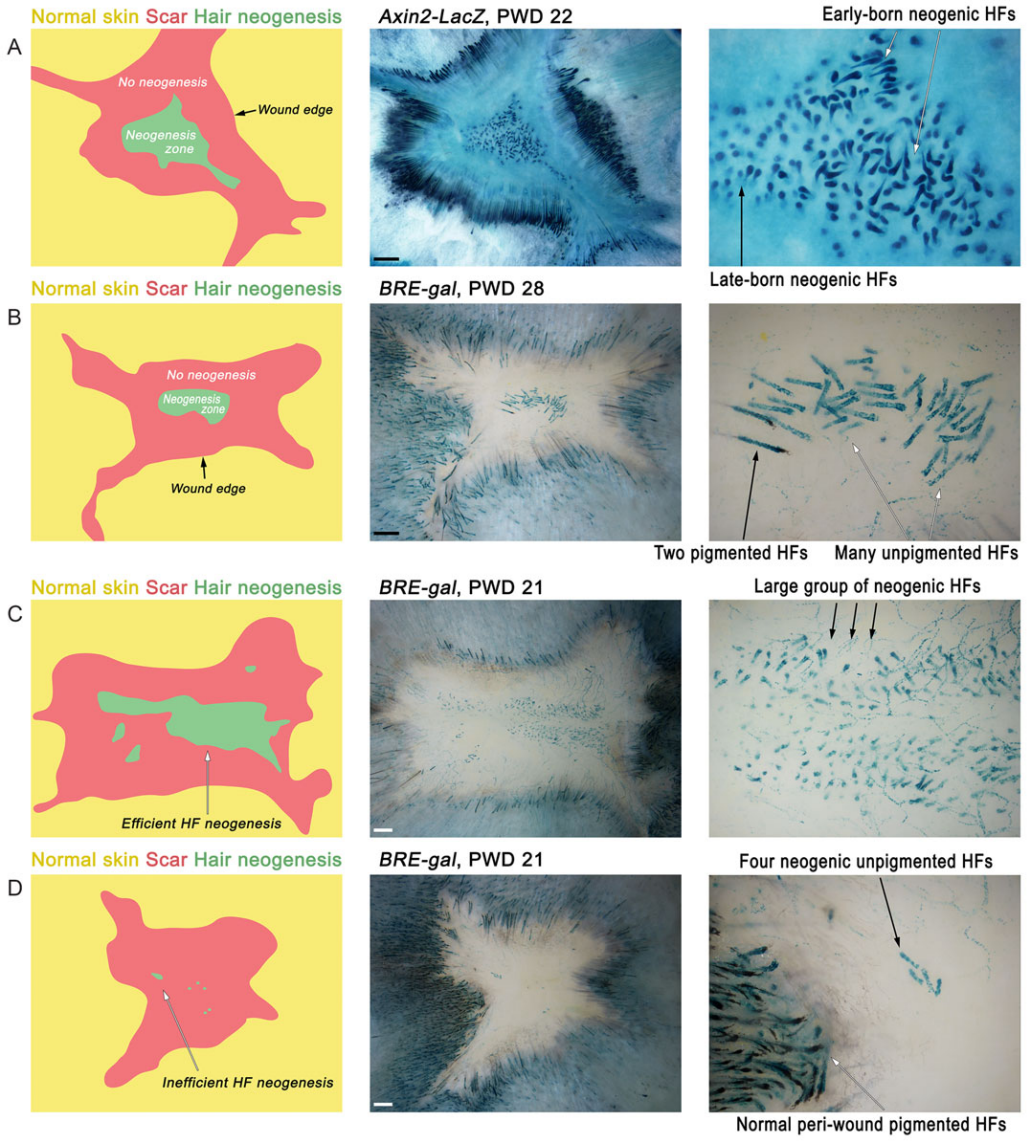
Key features of regenerated excisional skin wounds. (A) Typically, de novo HFs occupy the center of the regenerated wound (neogenesis zone, green). The neogenesis zone is always separated from the unwounded skin (yellow) by a hairless scar (red). This way, de novo HFs can be positively identified as residing in hair‐bearing areas surrounded by the rim of hairless scar tissue. At early PWD time points, both less and more mature de novo HFs can be seen, reflecting partial asynchrony of hair neogenesis. (B) Mature anagen de novo HFs are present during the late post‐wounding time period, PWD28. While the majority of the de novo HFs lack pigmentation, occasionally a few pigmented follicles can regenerate. (C), (D) Hair neogenesis displays a notable degree of variability, ranging from just a few HFs (D) to several hundreds (C). Here, WNT pathway reporter *Axin2‐LacZ* (A) and BMP pathway reporter *BRE‐gal* (B−D) mice (Javier et al. 2012) were used to aid visualization of neogenic hairs as strongly lacZ‐positive. Size bar 1 mm.

In *Mus musculus*, WIHN is typically observed following large wounding on the lower back, when a circular region of skin, at least 1 cm in diameter, is excised (Ito et al. 2007). Wound size appears to be the key factor determining WIHN activation, with excisional wounds smaller than 1 cm generally failing to regenerate de novo HFs. In adult mice older than 2 months, wounds 1.5 cm in diameter are recommended for more efficient WIHN. Early post‐wounding events in the WIHN model are typical of all excisional wounds and include reepithelialization over newly formed granulation tissue. These processes culminate in full reepithelialization and scab detachment on post‐wounding day 13−14 (PWD13−14) (Fig. 2). This time point, also referred to as scab detachment day 0 (SD0) (Fan et al. 2011), coincides with the onset of HF neogenesis. Placodes of the first de novo follicles appear on day SD1 and continue to emerge asynchronously over the course of the following week, until the process plateaus at around PWD21. Indeed, as exemplified in Figure 3A, neogenic hairs at various stages of morphogenesis can be seen within the wound's center at PWD22. In vivo temporal dynamics of HF neogenesis in the WIHN model are comprehensively covered by Fan et al. (2011).

**Figure 4:**
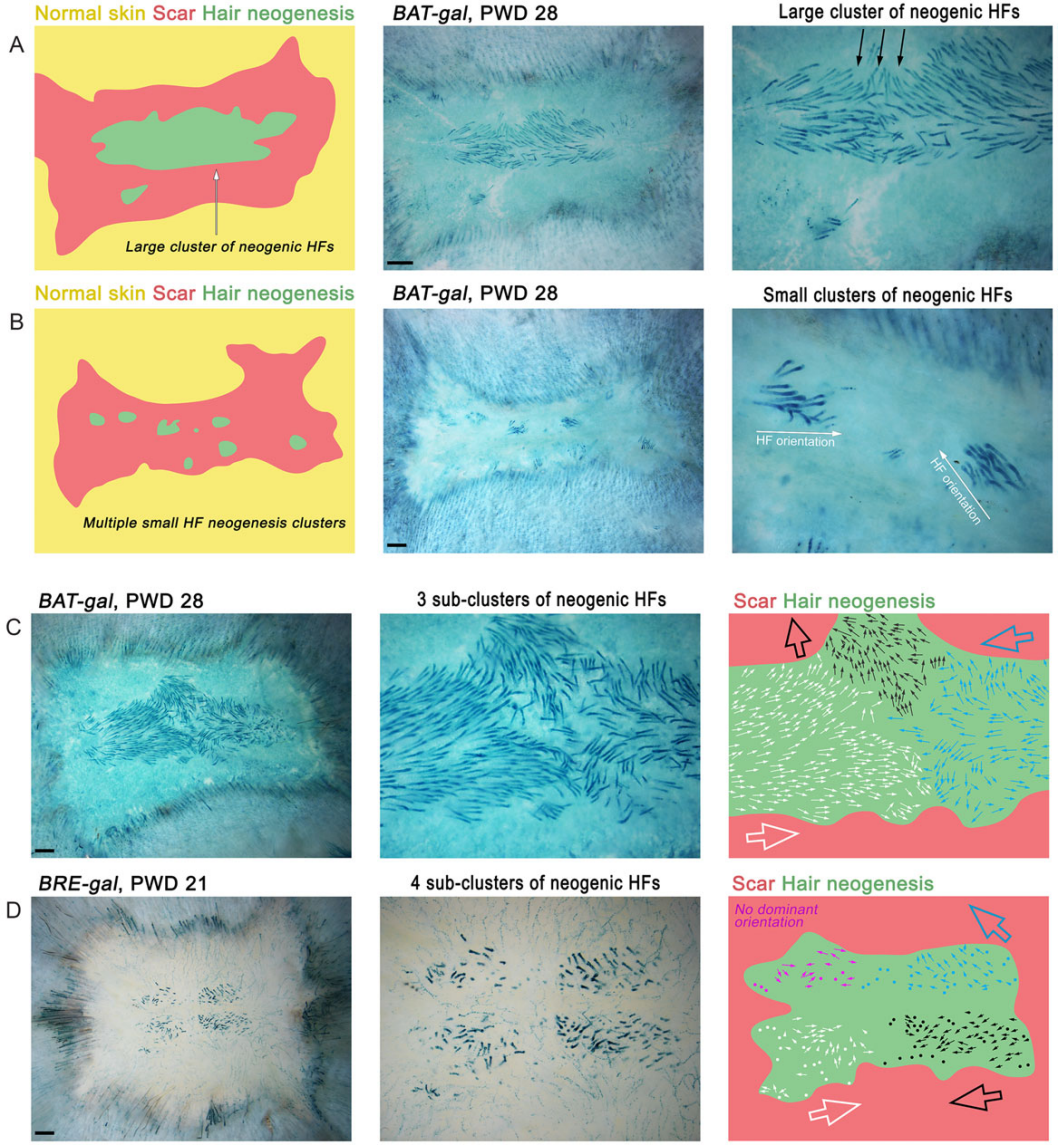
Distribution and orientation of neogenic hairs in regenerated wounds. (A) Commonly, regenerated HFs form one large cluster (also see Fig. 3). One or a few small secondary cluster(s) can also be present. (B) Seldom, multiple small de novo HF clusters can form (eight clusters here). (C), (D) Orientation of de novo HFs can range from seemingly random (D, purple region; also see Fig. 3) to unidirectional. Commonly, the neogenesis zone can contain several sub‐clusters of HFs with distinct orientation (C). Hairs have similar direction within a sub‐cluster, but often opposite of that in the neighboring sub‐cluster (white vs. black in C and D). Here, WNT pathway reporter *BAT‐gal* (A−C) and *BRE‐gal* reporters (D) were used to aid visualization of neogenic hairs as strongly lacZ‐positive. Size bar 1 mm.

Typically, de novo follicles form in the very center of the wound (Fig. 3A, B); however, more peripheral locations are not uncommon (Figs 3D, 4B). Importantly, in all instances, de novo follicles are separated from the preexisting follicles at the wound's edge by a circular, hairless scar (Fig. 3). All neogenic hairs have zigzag morphology (note that four distinct hair morphologies exist in normal mouse pelage: guard, awl, auchene, and zigzag [Sundberg & Hogan 1994]), and typically lack pigmentation in otherwise pigmented mice (Ito et al. 2007). In rare instances, a few pigmented neogenic hairs can also form (Fig. 3B). Commonly, de novo HFs form one large cluster with (Figs 3C, 4A) or without a few small satellite clusters (Fig. 3A, B). Rarely, multiple small clusters scattered throughout the wound can be observed (Fig. 4B). Importantly, even in age, gender, and strain matched littermates, the efficiency of hair neogenesis varies, ranging from just a few follicles (Fig. 3D) to several hundred (Fig. 3C), suggesting a stochastic component to the WIHN phenomenon. Classic accounts of WIHN in rabbits indicate that neogenesis can be very efficient with as many as 3500 de novo follicles forming per injured area (Billingham 1958) (note that neogenesis‐inducing wounds in rabbits are larger than in mice, usually 2.5 cm in diameter).

Importantly, de novo HFs contain functional bulge stem cells and undergo repetitive hair growth cycles, similar to normal HFs (Ito et al. 2007). Typically, de novo follicles enter first telogen at around PWD35 (albeit asynchronously due to their asynchronous morphogenesis), and then enter second anagen at around PWD45 (Fig. 2). Orientation is another important aspect of neogenic hairs. While normal hairs in the mouse dorsum follow the same cranial−caudal orientation (Guo et al. 2004), the axial position of neogenic hairs varies significantly. Although sometimes they appear to lack any specific orientation (Figs 3B and 4D, purple sub‐domain), they often share a common orientation within one cluster (Fig. 4B) or a sub‐cluster (Fig. 4A, C and D). The latter observation indicates that a rudimental hair patterning mechanism functions in the WIHN model.

## Cellular basis of de novo hair neogenesis—the possibility of a blastema‐like mechanism

Neogenic HFs in the wound's center regenerate all key epithelial and mesenchymal cell types characteristic of normal body hair. Critically, each neogenic follicle forms a new bulge populated by functional epithelial stem cells and a new mesenchymal dermal papilla (reviewed in Chuong 2007; Ito et al. 2007). Although the definitive origin of these de novo follicular cell populations remains to be elucidated, a few clues are beginning to emerge.

In their original study, Ito et al. (2007) elegantly showed that preexisting Krt15+ bulge stem cells in HFs located on the wound edge do not give rise to neogenic hairs. Although Krt15+ stem cells are efficiently recruited from peri‐wound HFs to the newly forming wound epidermis (Ito et al. 2005; reviewed in Plikus et al. 2012), their progeny are short‐lived and distinctly fail to contribute toward hair neogenesis. Therefore, de novo follicles do not regenerate from hair‐fated bulge epithelial progenitors. One possibility is that neogenic hairs regenerate from the progeny of Lrig1+ (Jensen et al. 2009), Lgr6+ (Snippert et al. 2010), and/or Gli1+ stem cells (Brownell et al. 2011) residing in the upper, supra‐bulge compartments of the peri‐wound follicles. Indeed, all of these stem/progenitor cell types were previously shown to give rise to long‐lasting cell clones in the wound epidermis (reviewed in Plikus et al. 2012). Another possibility is that progeny of bona fide interfollicular epidermal cells expand their lineage plasticity and acquire competence to regenerate hair lineages de novo—a level of cellular plasticity not normally observed in unwounded skin. The latter possibility implies some degree of epigenetic reprogramming in the wound epidermis and a blastema‐like mechanism for HF neogenesis. Although Shaw and Martin (2009) observed prominent changes in the epigenetic makeup of new wound epidermis, the overall mechanisms for epigenetic reprogramming during mouse wound healing remain largely unexplored (reviewed in Plikus et al. 2014).

Similarly, the lineage origin of neogenic dermal papillae in the WIHN model remains to be established. A recent study by Driskell et al. (2013) identified two principal dermal fibroblast lineages in the normal skin termed upper papillary and lower reticular populations. Lineage studies following wounding indicate that progeny of both reticular and papillary fibroblasts contribute to the wound's granulation tissue in successive waves. Although normal dermal papillae derive from the papillary lineage during embryonic hair morphogenesis, and papillary, but not reticular, fibroblasts display hair‐inducing properties in the so‐called chamber hair reconstitution assay (Driskell et al. 2013), future lineage studies are required to fully define the origin of neogenic dermal papillae in the WIHN model. At present, the blastema‐like mechanism, wherein wound fibroblasts undergo epigenetic reprogramming to a more plastic embryonic‐like state and acquire a dermal papilla identity via de novo lineage commitment, remains plausible.

Importantly, not all mesenchymal cell lineages appear to regenerate in the WIHN model. Billingham & Russell (1956) reported that, at least in rabbits, neogenic follicles lack the arrector pili muscle, which accompanies HFs in normal skin and whose contraction is responsible for the “goose bumps” effect. Future studies are required to comprehensively profile the types and the origin of all neogenic cell populations in the WIHN model.

## Signaling mechanism of de novo hair neogenesis

HF neogenesis critically depends on activation of the canonical WNT pathway, duplicating the signaling requirements of embryonic HF morphogenesis (Andl et al. 2002; Zhang et al. 2009). Ito et al. (2007) showed that ablation of WNT responsiveness in wound epidermis by means of inducible *β‐catenin* deletion completely ablated hair neogenesis. A similar effect was achieved via inducible overexpression of the WNT antagonist Dkk1 after, but not prior to, wound reepithelialization. Furthermore, overexpression of secreted WNT ligand throughout the wound epidermis in *Krt14‐Wnt7a* mice enhanced the efficiency of hair neogenesis by more than twofold. Correlations between WNT activation and HF neogenesis were also reported in the regenerating wounds of African spiny mice (Seifert et al. 2012).

What is the source(s) of WNT ligands that drives hair neogenesis in the WIHN model? A recent study by Myung et al. (2013) showed an essential role for epidermal WNTs. Inducible ablation of the WNT secreting function in the wound epidermis by means of *Wntless* (G‐protein‐coupled receptor 177) deletion in *Krt14‐CreER;Wls^fl/fl^* mice prevented regeneration of de novo hairs. In another study, Gay et al. (2013) showed an important role for dermal WNTs and also revealed the unexpected role of immune system cells in initiating the dermal WNT signaling cascade. They showed that WNT activation in WIHN is preceded by, and critically depends on, an earlier fibroblast growth factor 9 (Fgf9) signal generated by γδ T cells. Large numbers of γδ T cells migrate into the wound bed and proliferate a few days prior to the onset of HF neogenesis. They further showed that genetic ablation of γδ T cells or deletion of Fgf9 specifically in the T cell lineage decreased hair neogenesis efficiency. γδ T cell derived Fgf9 acts on the neighboring myofibroblasts in the early scar tissue, inducing them to secrete Wnt2a ligand for their subsequent WNT activation. Importantly, overexpression of WNT ligands in wound epidermis of the *Krt14‐Wnt7a* mice does not rescue hair neogenesis in mice lacking γδ T cells, suggesting largely non‐overlapping functions for dermal and epidermal WNT sources. The study by Gay et al. (2013) also showed that the signaling context of embryonic‐like regeneration can differ from that of actual embryonic morphogenesis. Development of normal HFs precedes maturation of T cells in embryogenesis, and although both morphogenetic processes probably depend upon the same signaling pathways, the source of signaling ligands can be different, thus providing regeneration with added flexibility.

Does hair neogenesis depend on the earlier, largely pro‐inflammatory signaling events that take place during the initial phase of wound closure? A recent study by Nelson et al. (2013) indicates a role for prostaglandin PGD2 signaling. Treatment of wounds with pro‐inflammatory PGD2 decreases the efficiency of hair neogenesis, while genetic deletion of the *Gpr44* PGD2 receptor increases it. Future studies on the WIHN model will be necessary to decipher how pro‐ and anti‐inflammatory signaling pathways act to suppress and/or augment pro‐regenerative WNT signaling.

## Unanswered questions and future challenges of the WIHN model

### Does cellular reprogramming take place?

The cellular basis for WIHN is not fully understood and two alternative mechanisms, stem cell versus reprogramming based, are yet to be exhaustively tested. According to the stem cell scenario, preexisting hair‐fated epithelial and dermal progenitors from wound edge HFs migrate towards the wound center and reassemble into de novo hairs. In a reprogramming scenario, dermal and/or epidermal cells undergo epigenetic “rewiring” towards a dedifferentiated, embryonic‐like state, followed by de novo redifferentiation towards hair‐fated lineages during WNT‐dependent HF neogenesis. Lineage studies by Ito et al. (2007) showed a lack of contribution to neogenic hairs from preexisting Krt15+ bulge stem cells, arguing against a stem cell based mechanism. The contribution from other supra‐bulge epithelial stem cells, Lrig1+ (Jensen et al. 2009), Lgr6+ (Snippert et al. 2010), and Gli1+ (Brownell et al. 2011), as well as from interfollicular epidermal basal layer cells remains to be comprehensively tested in the WIHN experiment. Likewise, contributions from hair‐fated dermal follicular cells, dermal papillae, and dermal sheath fibroblasts, as well as from diverse non‐hair‐fated dermal fibroblast types, await systematic testing. To that end, dermal‐cell‐specific *Cre* mouse lines are now undergoing characterization (Enshell‐Seijffers et al. 2010; Chen et al. 2012; Clavel et al. 2012; Hamburg & Atit 2012; Driskell et al. 2013; Fu & Hsu 2013; Rahmani et al. 2014), making such experiments technically feasible. Future lineage studies and in‐depth enquiries into the epigenetic state of dermal and epidermal wound cells will help to establish if in vivo cell fate reprogramming occurs in the WIHN model.

### Why does hair neogenesis occur within a narrow time window?

In the WIHN model in *Mus musculus*, neogenic hairs appear within a fairly narrow time window, between PWD14 and PWD21 (Ito et al. 2005; Fan et al. 2011), indicating that the conditions for de novo hair morphogenesis, in terms of cellular plasticity and/or inductive signaling, are temporally restricted. Indeed, if hair neogenesis does not occur within that window, a hairless scar results. Gay et al. (2013) established that the infiltration of Fgf9‐producing γδ T cells into the wound bed starts days before the onset of hair neogenesis, suggesting that this event inaugurates the hair neogenesis temporal window. Future profiling studies of cellular, signaling, and epigenetic dynamics around the time of hair neogenesis are likely to reveal additional pro‐regenerative factors that are time‐restricted.

### Why is hair neogenesis restricted to the wound's center?

Another aspect of hair neogenesis that remains to be understood is its spatial restriction within the wound center. Indeed, even in the instances when de novo hair formation is very efficient (Fig. 4C), the neogenesis zone is sharply demarcated from the wound's edge by a circular region of hairless scar. This feature is in contrast to mammalian digit tip regeneration. In the latter model, which distinctly relies on fate‐restricted progenitors (Lehoczky et al. 2011; Rinkevich et al. 2011), no scar is observed between the original and regenerated tissues. Assuming that WIHN relies on a reprogramming mechanism, it is possible that cells acquire greater lineage plasticity only in the wound's center. Another possibility is that pro‐regenerative signaling conditions, such as high WNT signaling, are spatially restricted. Yet another possibility is that the wound edge elicits “inhibitory” factors. Future studies are required to differentiate between these possibilities.

### What is the patterning mechanism of hair neogenesis?

Furthermore, several patterning features of the WIHN model provide a platform for studying principles and mechanisms of robustness during regeneration. Indeed, hair neogenesis is not as robust as normal embryonic hair development. Neogenesis, in terms of the number and patterning of de novo HFs in the wound, varies significantly even in age, gender, and strain matched littermates. It is well established that initiation of normal hair development relies on a reaction−diffusion patterning principle operating in the WNT‐dependent morphogenetic signaling field of the embryonic skin (Maini et al. 2006; Mou et al. 2006; Sick et al. 2006; Kondo & Miura 2010). Furthermore, the initial hair pattern is refined and augmented with the advent of secondary and tertiary HFs via the space‐filling expansion−induction mechanism, driven by the constant physical growth of embryonic skin (Cheng et al. 2014). Future studies will be required to understand which components of the reaction−diffusion and expansion−induction mechanisms, and/or other patterning mechanisms, operate in WIHN. Importantly, unlike embryonic skin, wounds do not expand in size following closure, probably preventing a mechanism similar to expansion−induction from activating.

Hair orientation is another patterning feature that differs between normal skin and WIHN. During normal skin development, anterior−posterior polarity is in place at the early hair germ stage via planar cell polarity (PCP) dependent mechanisms (Guo et al. 2004; Devenport & Fuchs 2008; Chang & Nathans 2013; Hua et al. 2014). Hair germs in PCP mutants, such as *Frizzled6* (Guo et al. 2004) or *Vangl2* loss‐of‐function mice (Devenport & Fuchs 2008), have a largely random orientation. Importantly, *Frizzled6* mutant HFs can reorient themselves locally via a non‐PCP‐dependent mechanism (Wang et al. 2006; 2010; Chang & Nathans 2013). As a result, initially random hair orientation converts into locally ordered hair domains, often leading to swirls and cross‐like patterns. Interestingly, neogenic HFs in WIHN often share common orientation within a domain (Fig. 4D), and when domains are sufficiently large, cross‐like patterns (Fig. 4C) reminiscent of those in *Frizzled6* mutants can arise in wild type wounds. Taken together, these correlations imply that WIHN fails to activate a global PCP‐dependent polarity mechanism. However, local PCP‐independent mechanisms may remain functional. Because the signaling nature of the PCP‐independent local hair reorientation process remains elusive (Wang et al. 2010; Chang & Nathans 2013), WIHN may prove to be a useful model for elucidating it.

Taken together, WIHN has emerged as the leading model system for studying the principles and mechanisms of embryonic‐like regeneration in mammals. WIHN features clear, quantifiable, and pattern‐forming regenerative outcomes, and the regenerative events follow a predictable and reproducible timeline. Multiple skin‐specific genetic tools and relatively well‐defined progenitor populations can provide the resources for targeted and in‐depth enquiries into the cellular, signaling, and epigenetic mechanisms of regeneration.

## References

[reg238-bib-0001] Andl, T. , Reddy, S.T. , Gaddapara, T. & Millar, S.E. (2002). WNT signals are required for the initiation of hair follicle development. Dev Cell, 2, 643–653. 12015971 10.1016/s1534-5807(02)00167-3

[reg238-bib-0002] Billingham, R.E. (1958). A reconsideration of the phenomenon of hair neogenesis, with particular reference to the healing of cutaneous wounds in adult mammals In: The Biology of Hair Growth, eds Montagna W. & Ellis R.A. Academic Press New York, NY, pp. 451–468.

[reg238-bib-0003] Billingham, R.E. & Russell, P.S. (1956). Incomplete wound contracture and the phenomenon of hair neogenesis in rabbits’ skin. Nature, 177, 791–792. 13321965 10.1038/177791b0

[reg238-bib-0004] Breedis, C. (1954). Regeneration of hair follicles and sebaceous glands from the epithelium of scars in the rabbit. Cancer Res, 14, 575–579. 13199800

[reg238-bib-0005] Brook, A.H. , Short, B.F. & Lyne, A.G. (1960). Formation of new wool follicles in the adult sheep. Nature, 185, 51. 13804752 10.1038/185051a0

[reg238-bib-0006] Brownell, I. , Guevara, E. , Bai, C.B. , Loomis, C.A. & Joyner, A.L. (2011). Nerve‐derived sonic hedgehog defines a niche for hair follicle stem cells capable of becoming epidermal stem cells. Cell Stem Cell, 8, 552–565. 21549329 10.1016/j.stem.2011.02.021 PMC3089905

[reg238-bib-0007] Chang, H. & Nathans, J. (2013). Responses of hair follicle‐associated structures to loss of planar cell polarity signaling. Proc Natl Acad Sci U S A, 110, E908–917. 23431170 10.1073/pnas.1301430110 PMC3593913

[reg238-bib-0008] Chen, D. , Jarrell, A. , Guo, C. , Lang, R. & Atit, R. (2012). Dermal beta‐catenin activity in response to epidermal Wnt ligands is required for fibroblast proliferation and hair follicle initiation. Development, 139, 1522–1533. 22434869 10.1242/dev.076463 PMC3308184

[reg238-bib-0009] Cheng, C.W. , Niu, B. , Warren, M. , Pevny, L.H. , Lovell‐Badge, R. , Hwa, T. et al. (2014). Predicting the spatiotemporal dynamics of hair follicle patterns in the developing mouse. Proc Natl Acad Sci U S A, 111, 2596–2601. 24550288 10.1073/pnas.1313083111 PMC3932898

[reg238-bib-0011] Chi, W.Y. , Enshell‐Seijffers, D. & Morgan, B.A. (2010). De novo production of dermal papilla cells during the anagen phase of the hair cycle. J Invest Dermatol, 130, 2664–2666. 20574444 10.1038/jid.2010.176 PMC2946972

[reg238-bib-0010] Chi, W. , Wu, E. & Morgan, B.A. (2013). Dermal papilla cell number specifies hair size, shape and cycling and its reduction causes follicular decline. Development, 140, 1676–1683. 23487317 10.1242/dev.090662 PMC3621486

[reg238-bib-0012] Christensen, R.N. , Weinstein, M. & Tassava, R.A. (2002). Expression of fibroblast growth factors 4, 8, and 10 in limbs, flanks, and blastemas of Ambystoma. Dev Dyn 223, 193–203. 11836784 10.1002/dvdy.10049

[reg238-bib-0013] Chuong, C.M. (2007). Regenerative biology: new hair from healing wounds. Nature, 447, 265–266. 17507966 10.1038/447265a PMC4377231

[reg238-bib-0014] Clavel, C. , Grisanti, L. , Zemla, R. , Rezza, A. , Barros, R. , Sennett, R. , et al. (2012). Sox2 in the dermal papilla niche controls hair growth by fine‐tuning BMP signaling in differentiating hair shaft progenitors. Dev Cell, 23, 981–994. 23153495 10.1016/j.devcel.2012.10.013 PMC3500526

[reg238-bib-0015] Cotsarelis, G. , Sun, T.T. & Lavker, R.M. (1990). Label‐retaining cells reside in the bulge area of pilosebaceous unit: implications for follicular stem cells, hair cycle, and skin carcinogenesis. Cell, 61, 1329–1337. 2364430 10.1016/0092-8674(90)90696-c

[reg238-bib-0016] Dann, L. , Glücksmann, A. & Tansley, K. (1941). The Healing of untreated experimental wounds. British Journal of Experimental Pathology, 22, 1–9.

[reg238-bib-0018] Desai, T.J. & Krasnow, M.A. (2013). Stem cells: differentiated cells in a back‐up role. Nature, 503, 204–205. 24196710 10.1038/nature12706 PMC4049315

[reg238-bib-0017] Desai, T.J. , Brownfield, D.G. & Krasnow, M.A. (2014). Alveolar progenitor and stem cells in lung development, renewal and cancer. Nature, 507, 190–194. 24499815 10.1038/nature12930 PMC4013278

[reg238-bib-0019] Devenport, D. & Fuchs, E. (2008). Planar polarization in embryonic epidermis orchestrates global asymmetric morphogenesis of hair follicles. Nat Cell Biol, 10, 1257–1268. 18849982 10.1038/ncb1784 PMC2607065

[reg238-bib-0020] Driskell, R.R. , Lichtenberger, B.M. , Hoste, E. , Kretzschmar, K. , Simons, B.D. , Charalambous, M. , et al. (2013). Distinct fibroblast lineages determine dermal architecture in skin development and repair. Nature, 504, 277–281. 24336287 10.1038/nature12783 PMC3868929

[reg238-bib-0021] Echeverri, K. & Tanaka, E.M. (2002). Ectoderm to mesoderm lineage switching during axolotl tail regeneration. Science, 298, 1993–1996. 12471259 10.1126/science.1077804

[reg238-bib-0022] Eguchi, G. , Abe, S.I. & Watanabe, K. (1974). Differentiation of lens‐like structures from newt iris epithelial cells in vitro. Proc Natl Acad Sci U S A, 71, 5052–5056. 4216028 10.1073/pnas.71.12.5052 PMC434038

[reg238-bib-0023] Enshell‐Seijffers, D. , Lindon, C. , Kashiwagi, M. & Morgan, B.A. (2010). Beta‐catenin activity in the dermal papilla regulates morphogenesis and regeneration of hair. Dev Cell, 18, 633–642. 20412777 10.1016/j.devcel.2010.01.016 PMC2893731

[reg238-bib-0024] Fan, C. , Luedtke, M.A. , Prouty, S.M. , Burrows, M. , Kollias, N. & Cotsarelis, G. (2011). Characterization and quantification of wound‐induced hair follicle neogenesis using in vivo confocal scanning laser microscopy. Skin Res Technol, 17, 387–397. 21492240 10.1111/j.1600-0846.2011.00508.x PMC3164919

[reg238-bib-0025] Freeman, G. (1963). Lens regeneration from the Cornea in Xenopus Laevis. J Exp Zool, 154, 39–65. 14066571 10.1002/jez.1401540105

[reg238-bib-0026] Fu, J. & Hsu, W. (2013). Epidermal Wnt controls hair follicle induction by orchestrating dynamic signaling crosstalk between the epidermis and dermis. J Invest Dermatol, 133, 890–898. 23190887 10.1038/jid.2012.407 PMC3594635

[reg238-bib-0027] Fuchs, Y. , Brown, S. , Gorenc, T. , Rodriguez, J. , Fuchs, E. & Steller, H. (2013). Sept4/ARTS regulates stem cell apoptosis and skin regeneration. Science, 341, 286–289. 23788729 10.1126/science.1233029 PMC4358763

[reg238-bib-0028] Gardiner, D.M. , Muneoka, K. & Bryant, S.V. (1986). The migration of dermal cells during blastema formation in axolotls. Dev Biol, 118, 488–493. 3792618 10.1016/0012-1606(86)90020-5

[reg238-bib-0029] Gay, D. , Kwon, O. , Zhang, Z. , Spata, M. , Plikus, M.V. , Holler, P.D. , et al. (2013). Fgf9 from dermal gammadelta T cells induces hair follicle neogenesis after wounding. Nat Med, 19, 916–923. 23727932 10.1038/nm.3181 PMC4054871

[reg238-bib-0030] Ghosh, S. , Roy, S. , Seguin, C. , Bryant, S.V. & Gardiner, D.M. (2008). Analysis of the expression and function of Wnt‐5a and Wnt‐5b in developing and regenerating axolotl (*Ambystoma mexicanum*) limbs. Dev Growth Differ, 50, 289–297. 18336582 10.1111/j.1440-169X.2008.01000.x

[reg238-bib-0031] Guo, N. , Hawkins, C. & Nathans, J. (2004). Frizzled6 controls hair patterning in mice. Proc Natl Acad Sci U S A, 101, 9277–9281. 15169958 10.1073/pnas.0402802101 PMC438967

[reg238-bib-0032] Hamburg, E.J. & Atit, R.P. (2012). Sustained beta‐catenin activity in dermal fibroblasts is sufficient for skin fibrosis. J Invest Dermatol, 132, 2469–2472. 22622416 10.1038/jid.2012.155 PMC3430808

[reg238-bib-0033] Hay, E.D. & Fischman, D.A. (1961). Origin of the blastema in regenerating limbs of the newt *Triturus viridescens*. An autoradiographic study using tritiated thymidine to follow cell proliferation and migration. Dev Biol, 3, 26–59. 13712434 10.1016/0012-1606(61)90009-4

[reg238-bib-0034] Henry, J.J. & Elkins, M.B. (2001). Cornea‐lens transdifferentiation in the anuran, *Xenopus tropicalis* . Dev Genes Evol, 211, 377–387. 11685571 10.1007/s004270100163

[reg238-bib-0035] Hirata, A. , Gardiner, D.M. & Satoh, A. (2010). Dermal fibroblasts contribute to multiple tissues in the accessory limb model. Dev Growth Differ, 52, 343–350. 20148925 10.1111/j.1440-169X.2009.01165.x

[reg238-bib-0036] Hsu, Y.C. , Pasolli, H.A. & Fuchs, E. (2011). Dynamics between stem cells, niche, and progeny in the hair follicle. Cell, 144, 92–105. 21215372 10.1016/j.cell.2010.11.049 PMC3050564

[reg238-bib-0037] Hua, Z.L. , Chang, H. , Wang, Y. , Smallwood, P.M. & Nathans, J. (2014). Partial interchangeability of Fz3 and Fz6 in tissue polarity signaling for epithelial orientation and axon growth and guidance. Development, 141, 3944–3954. 25294940 10.1242/dev.110189 PMC4197693

[reg238-bib-0038] Ito, M. , Liu, Y. , Yang, Z. , Nguyen, J. , Liang, F. , Morris, R.J. et al. (2005). Stem cells in the hair follicle bulge contribute to wound repair but not to homeostasis of the epidermis. Nat Med, 11, 1351–1354. 16288281 10.1038/nm1328

[reg238-bib-0039] Ito, M. , Yang, Z. , Andl, T. , Cui, C. , Kim, N. , Millar, S.E. et al. (2007). Wnt‐dependent de novo hair follicle regeneration in adult mouse skin after wounding. Nature, 447, 316–320. 17507982 10.1038/nature05766

[reg238-bib-0040] Jahoda, C.A. (2003). Cell movement in the hair follicle dermis—more than a two‐way street? J Invest Dermatol, 121, ix−xi. 10.1111/j.1523-1747.2003.12585.x 14675219

[reg238-bib-0041] Jahoda, C.A. , Oliver, R.F. , Reynolds, A.J. , Forrester, J.C. & Horne, K.A. (1996). Human hair follicle regeneration following amputation and grafting into the nude mouse. J Invest Dermatol, 107, 804–807. 8941664 10.1111/1523-1747.ep12330565

[reg238-bib-0042] Javier, A.L. , Doan, L.T. , Luong, M. , Reyes de Mochel, N.S. , Sun, A. , Monuki, E.S. et al. (2012). Bmp indicator mice reveal dynamic regulation of transcriptional response. PLoS One, 7, e42566. 22984405 10.1371/journal.pone.0042566 PMC3439458

[reg238-bib-0043] Jensen, K.B. , Collins, C.A. , Nascimento, E. , Tan, D.W. , Frye, M. , Itami, S. et al. (2009). Lrig1 expression defines a distinct multipotent stem cell population in mammalian epidermis. Cell Stem Cell, 4, 427–439. 19427292 10.1016/j.stem.2009.04.014 PMC2698066

[reg238-bib-0044] Kligman, A.M. (1959). Neogenesis of human hair follicles. Ann N Y Acad Sci, 83, 507–511. 14409843 10.1111/j.1749-6632.1960.tb40924.x

[reg238-bib-0045] Kligman, A.M. & Strauss, J.S. (1956). The formation of vellus hair follicles from human adult epidermis. J Invest Dermatol, 27, 19–23. 13357817 10.1038/jid.1956.71

[reg238-bib-0046] Kondo, S. & Miura, T. (2010). Reaction‐diffusion model as a framework for understanding biological pattern formation. Science, 329, 1616–1620. 20929839 10.1126/science.1179047

[reg238-bib-0047] Kragl, M. , Knapp, D. , Nacu, E. , Khattak, S. , Maden, M. , Epperlein, H.H. et al. (2009). Cells keep a memory of their tissue origin during axolotl limb regeneration. Nature, 460, 60–65. 19571878 10.1038/nature08152

[reg238-bib-0048] Lehoczky, J.A. , Robert, B. & Tabin, C.J. (2011). Mouse digit tip regeneration is mediated by fate‐restricted progenitor cells. Proc Natl Acad Sci U S A, 108, 20609–20614. 22143790 10.1073/pnas.1118017108 PMC3251149

[reg238-bib-0049] Leung, Y. , Kandyba, E. , Chen, Y.B. , Ruffins, S. , Chuong, C.M. & Kobielak, K. (2014). Bifunctional ectodermal stem cells around the nail display dual fate homeostasis and adaptive wounding response toward nail regeneration. Proc Natl Acad Sci U S A, 111, 15114–15119. 25277970 10.1073/pnas.1318848111 PMC4210315

[reg238-bib-0050] Maini, P.K. , Baker, R.E. & Chuong, C.M. (2006). Developmental biology. The Turing model comes of molecular age. Science, 314, 1397–1398. 17138885 10.1126/science.1136396 PMC4383235

[reg238-bib-0051] Makanae, A. , Mitogawa, K. & Satoh, A. (2014). Co‐operative Bmp‐ and Fgf‐signaling inputs convert skin wound healing to limb formation in urodele amphibians. Dev Biol, 396, 57–66. 25286122 10.1016/j.ydbio.2014.09.021

[reg238-bib-0052] McCusker, C.D. & Gardiner, D.M. (2013). Positional information is reprogrammed in blastema cells of the regenerating limb of the axolotl (*Ambystoma mexicanum*). PLoS One, 8, e77064. 24086768 10.1371/journal.pone.0077064 PMC3785456

[reg238-bib-0053] McElwee, K.J. , Kissling, S. , Wenzel, E. , Huth, A. & Hoffmann, R. (2003). Cultured peribulbar dermal sheath cells can induce hair follicle development and contribute to the dermal sheath and dermal papilla. J Invest Dermatol, 121, 1267–1275. 14675169 10.1111/j.1523-1747.2003.12568.x

[reg238-bib-0054] Mikhail, G.R. (1963). Hair Neogenesis in Rat Skin. Arch Dermatol, 88, 713–728. 14071440 10.1001/archderm.1963.01590240037008

[reg238-bib-0055] Morris, R.J. , Liu, Y. , Marles, L. , Yang, Z. , Trempus, C. , Li, S. , et al. (2004). Capturing and profiling adult hair follicle stem cells. Nat Biotechnol, 22, 411–417. 15024388 10.1038/nbt950

[reg238-bib-0056] Mou, C. , Jackson, B. , Schneider, P. , Overbeek, P.A. & Headon, D.J. (2006). Generation of the primary hair follicle pattern. Proc Natl Acad Sci U S A, 103, 9075–9080. 16769906 10.1073/pnas.0600825103 PMC1482568

[reg238-bib-0057] Muneoka, K. , Fox, W.F. & Bryant, S.V. (1986). Cellular contribution from dermis and cartilage to the regenerating limb blastema in axolotls. Dev Biol, 116, 256–260. 3732605 10.1016/0012-1606(86)90062-x

[reg238-bib-0058] Myung, P.S. , Takeo, M. , Ito, M. & Atit, R.P. (2013). Epithelial Wnt ligand secretion is required for adult hair follicle growth and regeneration. J Invest Dermatol, 133, 31–41. 22810306 10.1038/jid.2012.230 PMC3479363

[reg238-bib-0059] Nelson, A.M. , Loy, D.E. , Lawson, J.A. , Katseff, A.S. , Fitzgerald, G.A. & Garza, L.A. (2013). Prostaglandin D2 inhibits wound‐induced hair follicle neogenesis through the receptor, Gpr44. J Invest Dermatol, 133, 881–889. 23190891 10.1038/jid.2012.398 PMC3593761

[reg238-bib-0061] Oliver, R.F. (1966). Whisker growth after removal of the dermal papilla and lengths of follicle in the hooded rat. J Embryol Exp Morphol, 15, 331–347. 5964281

[reg238-bib-0062] Oliver, R.F. (1967). The experimental induction of whisker growth in the hooded rat by implantation of dermal papillae. J Embryol Exp Morphol, 18, 43–51. 6048979

[reg238-bib-0060] O'Steen, W.K. & Walker, B.E. (1961). Radioautographic studies of regeneration in the common newt. II. Regeneration of the forelimb. The Anatomical Record, 139, 547–555.

[reg238-bib-0063] Plikus, M.V. (2014). At the dawn of hair research—testing the limits of hair follicle regeneration. Exp Dermatol, 23, 314–315. 24494858 10.1111/exd.12334

[reg238-bib-0064] Plikus, M.V. , Gay, D.L. , Treffeisen, E. , Wang, A. , Supapannachart, R.J. & Cotsarelis, G. (2012). Epithelial stem cells and implications for wound repair. Semin Cell Dev Biol, 23, 946–953. 23085626 10.1016/j.semcdb.2012.10.001 PMC3518754

[reg238-bib-0065] Plikus, M.V. , Guerrero‐Juarez, C.F. , Treffeisen, E. & Gay, D.L. (2014). Epigenetic control of skin and hair regeneration after wounding. Exp Dermatol. 24, 167–170 25039994 10.1111/exd.12488 PMC4289120

[reg238-bib-0066] Rahmani, W. , Abbasi, S. , Hagner, A. , Raharjo, E. , Kumar, R. , Hotta, A. , et al. (2014). Hair follicle dermal stem cells regenerate the dermal sheath, repopulate the dermal papilla, and modulate hair type. Dev Cell, 31, 543–558. 25465495 10.1016/j.devcel.2014.10.022

[reg238-bib-0067] Rinkevich, Y. , Lindau, P. , Ueno, H. , Longaker, M.T. & Weissman, I.L. (2011). Germ‐layer and lineage‐restricted stem/progenitors regenerate the mouse digit tip. Nature, 476, 409–413. 21866153 10.1038/nature10346 PMC3812235

[reg238-bib-0068] Roensch, K. , Tazaki, A. , Chara, O. & Tanaka, E.M. (2013). Progressive specification rather than intercalation of segments during limb regeneration. Science, 342, 1375–1379. 24337297 10.1126/science.1241796

[reg238-bib-0069] Rompolas, P. , Deschene, E.R. , Zito, G. , Gonzalez, D.G. , Saotome, I. , Haberman, A.M. et al. (2012). Live imaging of stem cell and progeny behaviour in physiological hair‐follicle regeneration. Nature, 487, 496–499. 22763436 10.1038/nature11218 PMC3772651

[reg238-bib-0070] Rompolas, P. , Mesa, K.R. & Greco, V. (2013). Spatial organization within a niche as a determinant of stem‐cell fate. Nature, 502, 513–518. 24097351 10.1038/nature12602 PMC3895444

[reg238-bib-0071] Sandoval‐Guzman, T. , Wang, H. , Khattak, S. , Schuez, M. , Roensch, K. , Nacu, E. , et al. (2014). Fundamental differences in dedifferentiation and stem cell recruitment during skeletal muscle regeneration in two salamander species. Cell Stem Cell, 14, 174–187. 24268695 10.1016/j.stem.2013.11.007

[reg238-bib-0072] Satoh, A. , Bryant, S.V. & Gardiner, D.M. (2008a). Regulation of dermal fibroblast dedifferentiation and redifferentiation during wound healing and limb regeneration in the Axolotl. Dev Growth Differ, 50, 743–754. 19046162 10.1111/j.1440-169X.2008.01072.x

[reg238-bib-0073] Satoh, A. , Graham, G.M. , Bryant, S.V. & Gardiner, D.M. (2008b). Neurotrophic regulation of epidermal dedifferentiation during wound healing and limb regeneration in the axolotl (*Ambystoma mexicanum*). Dev Biol, 319, 321–335. 18533144 10.1016/j.ydbio.2008.04.030

[reg238-bib-0074] Satoh, A. , Makanae, A. , Hirata, A. & Satou, Y. (2011). Blastema induction in aneurogenic state and Prrx‐1 regulation by MMPs and FGFs in *Ambystoma mexicanum* limb regeneration. Dev Biol, 355, 263–274. 21539833 10.1016/j.ydbio.2011.04.017

[reg238-bib-0075] Schneider, M.R. , Schmidt‐Ullrich, R. & Paus, R. (2009). The hair follicle as a dynamic miniorgan. Curr Biol, 19, R132–142. 19211055 10.1016/j.cub.2008.12.005

[reg238-bib-0077] Seifert, A.W. & Maden, M. (2014). New insights into vertebrate skin regeneration. Int Rev Cell Mol Biol, 310, 129–169. 24725426 10.1016/B978-0-12-800180-6.00004-9

[reg238-bib-0076] Seifert, A.W. , Kiama, S.G. , Seifert, M.G. , Goheen, J.R. , Palmer, T.M. et al. (2012). Skin shedding and tissue regeneration in African spiny mice (*Acomys*). Nature, 489, 561–565. 23018966 10.1038/nature11499 PMC3480082

[reg238-bib-0078] Shaw, T. & Martin, P. (2009). Epigenetic reprogramming during wound healing: loss of polycomb‐mediated silencing may enable upregulation of repair genes. EMBO Rep, 10, 881–886. 19575012 10.1038/embor.2009.102 PMC2726669

[reg238-bib-0079] Shimokawa, T. , Yasutaka, S. , Kominami, R. & Shinohara, H. (2013). Lmx‐1b and Wnt‐7a expression in axolotl limb during development and regeneration. Okajimas Folia Anat Jpn, 89, 119–124. 23614984 10.2535/ofaj.89.119

[reg238-bib-0080] Sick, S. , Reinker, S. , Timmer, J. & Schlake, T. (2006). WNT and DKK determine hair follicle spacing through a reaction‐diffusion mechanism. Science, 314, 1447–1450. 17082421 10.1126/science.1130088

[reg238-bib-0081] Singer, M. & Inoue, S. (1964). The Nerve and the Epidermal Apical Cap in Regeneration of the Forelimb of Adult Triturus. J Exp Zool, 155, 105–116. 14118835 10.1002/jez.1401550108

[reg238-bib-0082] Snippert, H.J. , Haegebarth, A. , Kasper, M. , Jaks, V. , van Es, J.H. , Barker, N. , et al. (2010). Lgr6 marks stem cells in the hair follicle that generate all cell lineages of the skin. Science, 327, 1385–1389. 20223988 10.1126/science.1184733

[reg238-bib-0083] Stange, D.E. , Koo, B.K. , Huch, M. , Sibbel, G. , Basak, O. , Lyubimova, A. , et al. (2013). Differentiated Troy+ chief cells act as reserve stem cells to generate all lineages of the stomach epithelium. Cell, 155, 357–368. 24120136 10.1016/j.cell.2013.09.008 PMC4094146

[reg238-bib-0084] Stenbäck, F. , Niinimäki, T. & Dammert, K. (1967). Hair neogenesis in rat and rabbit skin. Acta Pathologica Microbiologica Scandinavica, 69, 480.

[reg238-bib-0085] Stenn, K.S. & Paus, R. (2001). Controls of hair follicle cycling. Physiol Rev, 81, 449–494. 11152763 10.1152/physrev.2001.81.1.449

[reg238-bib-0086] Straile, W.E. (1959). A study on the neoformation of mammalian hair follicles. Ann N Y Acad Sci, 83, 499–506. 13835059 10.1111/j.1749-6632.1960.tb40923.x

[reg238-bib-0087] Sun, G. , Zhang, X. , Shen, Y.I. , Sebastian, R. , Dickinson, L.E. , Fox‐Talbot, K. , et al. (2011). Dextran hydrogel scaffolds enhance angiogenic responses and promote complete skin regeneration during burn wound healing. Proc Natl Acad Sci U S A, 108, 20976–20981. 22171002 10.1073/pnas.1115973108 PMC3248550

[reg238-bib-0088] Sundberg, J.P. & Hogan, M.E. (1994). Hair types and subtypes in the laboratory mouse. In: Handbook of mouse mutations with skin and hair abnormalities, eds Sundberg J.P. CRC Press, Boca Raton, FL, pp. 57–68.

[reg238-bib-0089] Takeo, M. , Chou, W.C. , Sun, Q. , Lee, W. , Rabbani, P. , Loomis, C. , et al. (2013). Wnt activation in nail epithelium couples nail growth to digit regeneration. Nature, 499, 228–232. 23760480 10.1038/nature12214 PMC3936678

[reg238-bib-0090] Takeo, M. , Lee, W. & Ito, M. (2015). Wound Healing and Skin Regeneration. Cold Spring Harb Perspect Med, 5, a023267. 10.1101/cshperspect.a023267 PMC4292081 25561722

[reg238-bib-0091] Tanaka, E.M. (2012). Regenerative biology: skin, heal thyself. Nature, 489, 508–510. 23018959 10.1038/489508a

[reg238-bib-0092] Tata, P.R. , Mou, H. , Pardo‐Saganta, A. , Zhao, R. , Prabhu, M. , Law, B.M. , et al. (2013). Dedifferentiation of committed epithelial cells into stem cells in vivo. Nature, 503, 218–223. 24196716 10.1038/nature12777 PMC4035230

[reg238-bib-0093] Taylor, A.C. (1949). Survival of rat skin and changes in hair pigmentation following freezing. J Exp Zool, 110, 77–111. 18113442 10.1002/jez.1401100106

[reg238-bib-0094] Taylor, G. , Lehrer, M.S. , Jensen, P.J. , Sun, T.T. & Lavker, R.M. (2000). Involvement of follicular stem cells in forming not only the follicle but also the epidermis. Cell, 102, 451–461. 10966107 10.1016/s0092-8674(00)00050-7

[reg238-bib-0095] Tobin, D.J. , Gunin, A. , Magerl, M. , Handijski, B. & Paus, R. (2003). Plasticity and cytokinetic dynamics of the hair follicle mesenchyme: implications for hair growth control. J Invest Dermatol, 120, 895–904. 12787113 10.1046/j.1523-1747.2003.12237.x

[reg238-bib-0096] Tsonis, P.A. & Del Rio‐Tsonis, K. (2004). Lens and retina regeneration: transdifferentiation, stem cells and clinical applications. Exp Eye Res, 78, 161–172. 14729349 10.1016/j.exer.2003.10.022

[reg238-bib-0097] Tumbar, T. , Guasch, G. , Greco, V. , Blanpain, C. , Lowry, W.E. , Rendl, M. et al. (2004). Defining the epithelial stem cell niche in skin. Science, 303, 359–363. 14671312 10.1126/science.1092436 PMC2405920

[reg238-bib-0098] Wang, Y. , Badea, T . & Nathans, J. (2006). Order from disorder: self‐organization in mammalian hair patterning. Proc Natl Acad Sci U S A, 103, 19800–19805. 17172440 10.1073/pnas.0609712104 PMC1750877

[reg238-bib-0099] Wang, Y. , Chang, H. & Nathans, J. (2010). When whorls collide: the development of hair patterns in frizzled 6 mutant mice. Development, 137, 4091–4099. 21062866 10.1242/dev.057455 PMC2976288

[reg238-bib-0100] Yanger, K. & Stanger, B.Z. (2014). Liver cell reprogramming: parallels with iPSC biology. Cell Cycle, 13, 1211–1212. 24621496 10.4161/cc.28381 PMC4049951

[reg238-bib-0101] Yanger, K. , Zong, Y. , Maggs, L.R. , Shapira, S.N. , Maddipati, R. , Aiello, N.M. , et al. (2013). Robust cellular reprogramming occurs spontaneously during liver regeneration. Genes Dev, 27, 719–724. 23520387 10.1101/gad.207803.112 PMC3639413

[reg238-bib-0102] Zhang, Y. , Tomann, P. , Andl, T. , Gallant, N.M. , Huelsken, J. , Jerchow, B. , et al. (2009). Reciprocal requirements for EDA/EDAR/NF‐kappaB and Wnt/beta‐catenin signaling pathways in hair follicle induction. Dev Cell, 17, 49–61. 19619491 10.1016/j.devcel.2009.05.011 PMC2859042

